# Automated recognition of malignancy mentions in biomedical literature

**DOI:** 10.1186/1471-2105-7-492

**Published:** 2006-11-07

**Authors:** Yang Jin, Ryan T McDonald, Kevin Lerman, Mark A Mandel, Steven Carroll, Mark Y Liberman, Fernando C Pereira, Raymond S Winters, Peter S White

**Affiliations:** 1 Department of Pediatrics, University of Pennsylvania, Philadelphia PA 19104 USA; 2 Department of Computer and Information Science, University of Pennsylvania, Philadelphia PA 19104 USA; 3 The Children's Hospital of Philadelphia, Philadelphia PA 19104 USA; 4 Linguistic Data Consortium, University of Pennsylvania, Philadelphia PA 19104 USA

## Abstract

**Background:**

The rapid proliferation of biomedical text makes it increasingly difficult for researchers to identify, synthesize, and utilize developed knowledge in their fields of interest. Automated information extraction procedures can assist in the acquisition and management of this knowledge. Previous efforts in biomedical text mining have focused primarily upon named entity recognition of well-defined molecular objects such as genes, but less work has been performed to identify disease-related objects and concepts. Furthermore, promise has been tempered by an inability to efficiently scale approaches in ways that minimize manual efforts and still perform with high accuracy. Here, we have applied a machine-learning approach previously successful for identifying molecular entities to a disease concept to determine if the underlying probabilistic model effectively generalizes to unrelated concepts with minimal manual intervention for model retraining.

**Results:**

We developed a named entity recognizer (MTag), an entity tagger for recognizing clinical descriptions of malignancy presented in text. The application uses the machine-learning technique Conditional Random Fields with additional domain-specific features. MTag was tested with 1,010 training and 432 evaluation documents pertaining to cancer genomics. Overall, our experiments resulted in 0.85 precision, 0.83 recall, and 0.84 F-measure on the evaluation set. Compared with a baseline system using string matching of text with a neoplasm term list, MTag performed with a much higher recall rate (92.1% vs. 42.1% recall) and demonstrated the ability to learn new patterns. Application of MTag to all MEDLINE abstracts yielded the identification of 580,002 unique and 9,153,340 overall mentions of malignancy. Significantly, addition of an extensive lexicon of malignancy mentions as a feature set for extraction had minimal impact in performance.

**Conclusion:**

Together, these results suggest that the identification of disparate biomedical entity classes in free text may be achievable with high accuracy and only moderate additional effort for each new application domain.

## Background

The biomedical literature collectively represents the acknowledged historical perception of biological and medical concepts, including findings pertaining to disease-related research. However, the rapid proliferation of this information makes it increasingly difficult for researchers and clinicians to peruse, query, and synthesize it for biomedical knowledge gain. Automated information extraction methods, which have recently been increasingly concentrated upon biomedical text, can assist in the acquisition and management of this data. Although text mining applications have been successful in other domains and show promise for biomedical information extraction, issues of scalability impose significant impediments to broad use in biomedicine. Particular challenges for text mining include the requirement for highly specified extractors in order to generate accuracies sufficient for users; considerable effort by highly trained computer scientists with substantial input by biomedical domain experts to develop extractors; and a significant body of manually annotated text – with comparable effort in generating annotated corpora – for training machine-learning extractors. In addition, the high number and wide diversity of biomedical entity types, along with the high complexity of biomedical literature, makes auto-annotation of multiple biomedical entity classes a difficult and labor-intensive task.

Most biomedical text mining efforts to date have focused upon molecular object (entity) classes, especially the identification of gene and protein names. Automated extractors for these tasks have improved considerably in the last few years [1-13]. We recently extended this focus to include genomic variations [14]. Although there have been efforts to apply automated entity recognition to the identification of phenotypic and disease objects [15-17], these systems are broadly focused and often do not perform as well as those utilizing more recently-evolved machine-learning techniques for such tasks as gene/protein name recognition. Recently, Skounakis and colleagues have applied a machine-learning algorithm to extract gene-disorder relations [18], while van Driel and co-workers have made attempts to extract phenotypic attributes from Online Mendelian Inheritance in Man [19]. However, more extensive work on medical entity class recognition is necessary because it is an important prerequisite for utilizing text information to link molecular and phenotypic observations, thus improving the association between laboratory research and clinical applications described in the literature.

In the current work, we explore scalability issues relating to entity extractor generality and development time, and also determine the feasibility of efficiently capturing disease descriptions. We first describe an algorithm for automatically recognizing a specific disease entity class: malignant disease labels. This algorithm, MTag, is based upon the probability model Conditional Random Fields (CRFs) that has been shown to perform with state-of-the-art accuracy for entity extraction tasks [5,14]. CRF extractors consider a large number of syntactic and semantic features of text surrounding each putative mention [20,21]. MTag was trained and evaluated on MEDLINE abstracts and compared with a baseline vocabulary matching method. An MTag output format that provides HTML-visualized markup of malignant mentions was developed. Finally, we applied MTag to the entire collection of MEDLINE abstracts to generate an annotated corpus and an extensive vocabulary of malignancy mentions.

## Results

### MTag performance

Manually annotated text from a corpus of 1,442 MEDLINE abstracts was used to train and evaluate MTag. Abstracts were derived from a random sampling of two domains: articles pertaining to the pediatric tumor neuroblastoma and articles describing genomic alterations in a wide variety of malignancies. Two separate training experiments were performed, either with or without the inclusion of malignancy-specific features, which were the addition of a lexicon of malignancy mentions and a list of indicative suffixes. In each case, MTag was tested with the same randomly selected 1,010 training documents and then evaluated with a separate set of 432 documents pertaining to cancer genomics. The extractor took approximately 6 hours to train on a 733 MHz PowerPC G4 with 1 GB SDRAM. Once trained, MTag can annotate a new abstract in a matter of seconds.

For evaluation purposes, manual annotations were treated as gold-standard files (assuming 100% annotation accuracy). We first evaluated the MTag model with all biological feature sets included. Our experiments resulted in 0.846 precision, 0.831 recall, and 0.838 F-measure on the evaluation set. Additionally, the two subset corpora (neuroblastoma-specific and genome-specific) were tested separately. As expected, the extractor performed with higher accuracy with the more narrowly defined corpus (neuroblastoma) than with the corpus more representative for various malignancies (genome-specific). The neuroblastoma corpus performed with 0.88 precision, 0.87 recall, and 0.88 F-measure, while the genome-specific corpus performed with 0.77 precision, 0.69 recall, and 0.73 F-measure. These results likely reflect the increased challenge of identifying mentions of malignancy in a document set demonstrating a more diverse collection of mentions.

Next, we excluded our biological feature sets from MTag to create a generic extractor, in order to determine the impact of these domain-specific features. This extractor was then trained and evaluated using the identical set of files used to train the biological MTag version. Somewhat surprisingly, the extractor performed with similar accuracy with the generic model, resulting in 0.851 precision, 0.818 recall, and 0.834 F-measure on the evaluation set. These results suggested that at least for this class of entities, the extractor performs the task of identifying malignancy mentions efficiently without the use of a specialized lexicon.

### Extraction versus string matching

We next determined performance of MTag relative to a baseline system that could be easily employed. For the baseline system, the NCI neoplasm ontology, a term list of 5,555 malignancies, was used as a lexicon to identify malignancy mentions [22]. Lexicon terms were individually queried against text by case-insensitive exact string matching. A subset of 39 abstracts randomly selected from the testing set, which together contained 202 malignancy mentions, were used to compare the automated extractor and baseline results. MTag identified 190 of the 202 mentions correctly (94.1%), while the NCI list identified only 85 mentions (42.1%), all of which were also identified by the extractor. We also determined the performance of string matching that instead used the set of malignancy mentions identified in the manually curated training set annotations (1,010 documents) as a matching lexicon. This system identified 79 of 202 mentions (39.1%). Combining the manually-derived lexicon with the NCI lexicon yielded 124 of 202 matches (61.4%).

A closer analysis of the 68 malignancy mentions missed by the string matching with combined lists but positively identified by MTag determined two general subclasses of additional malignant mentions. The majority of MTag-unique mentions were lexical or modified variations of malignancies present either in the training data or in the NCI lexicon, such as minor variations in spelling and form (e.g., "leukaemia" versus "leukemia"), and acronyms (e.g., "AML" in place of "acute myeloid leukemia"). More importantly, a substantial minority of mentions identified only by MTag were instances of the extractor determining new mentions of malignancies that were, in many cases, neither obvious nor represented in readily available lexicons. For example, "temporal lobe benign capillary haemangioblastoma" and "parietal lobe ganglioglioma" are neither in the NCI list or training set per se, or approximated as such by a lexical variant. This suggests that MTag contributes a significant learning component.

### Application to MEDLINE

MTag was then used to extract mentions of malignancy from all MEDLINE abstracts through 2005. Extraction took 1,642 CPU-hours (68.4 CPU-days; 2.44 days on our 28-CPU cluster) to process 15,433,668 documents. A total of 9,153,340 redundant mentions and 580,002 unique mentions (ignoring case) were identified. Interestingly, the ratio of unique new mentions identified relative to the number of abstracts analyzed was relatively uniform, ranging from a rate of 0.183 new mentions per abstract for the first 0.1% of documents to a rate of 0.038 new mentions per abstract for the last 1% of documents. This indicated that a substantial rate of new mentions was being maintained throughout the extraction process.

The 25 mentions found in the greatest number of abstracts by MTag are listed in Table 1. Six of these malignant phrases: pulmonary, fibroblasts, neoplastic, neoplasm metastasis, extramural, and abdominal did not match our definition of malignancy. Of these, only "extramural" is not frequently associated with malignancy descriptions and is likely the result of containing character n-grams that are generally indicative of malignancy mentions. The remaining five phrases are likely the result of the extractor failing to properly define mention boundaries in certain cases (e.g., tagging "neoplasm" rather than "brain neoplasm"), or alternatively, shared use of an otherwise indicative character string (e.g., "opl" in "brain neoplasm" and "neoplastic") between a true positive and a false positive.

**Table 1:** Top 25 MTag identified mentions and their corresponding PubMED keyword and MEDLINE exact string matching search results.

**MTag-identified Mentions**	**Evaluation**	**MTag articles**	**PubMED keyword articles**	**MEDLINE exact matches**
carcinoma	True Positive	861214	466958	891996
breast neoplasms	True Positive	129096	133592	137445
adenocarcinoma	True Positive	166302	208117	183654
lung neoplasms	True Positive	104176	110378	111869
pulmonary	False Positive			
breast cancer	True Positive	91446	147286	128381
lymphoma	True Positive	182764	158674	226407
liver neoplasms	True Positive	69513	84529	84712
fibroblasts	False Positive			
skin neoplasms	True Positive	62282	66072	66105
neoplastic	False Positive			
neoplasm metastasis	False Positive			
brain neoplasms	True Positive	58729	84636	63586
stomach neoplasms	True Positive	50019	52566	55208
prostatic neoplasms	True Positive	48042	49110	50312
leukemia	True Positive	163011	190798	368980
colonic neoplasms	True Positive	41327	47402	42841
cervical neoplasms	True Positive	40998	41424	41717
sarcoma	True Positive	142665	110920	242654
bone neoplasms	True Positive	33568	73429	35091
melanoma	True Positive	79519	61134	126681
pancreatic neoplasms	True Positive	31598	33775	33291
extramural	False Positive			
lung cancer	True Positive	53601	118679	66071
abdominal	False Positive			

For comparison, we also determined the corresponding number of articles identified both by keyword searching of PubMed and by exact string matching of MEDLINE for each of the 19 most common true malignancy types (Table 1). Overall, MTag's comparative recall was 1.076 versus PubMed keyword searching and 0.814 versus string matching. As PubMed keyword searching uses concept mapping to relate keywords to related concepts, thus providing query expansion, the document retrieval totals derived from this approach do not strictly compare to MTag's approach. Furthermore, the exact string totals would be inflated relative to the MTag totals, as for example the phrase "myeloid leukemia" would be counted both for this category and for a category "leukemia" with exact string matching, but would only be counted for the former phrase by MTag. To adjust for these discrepancies, for MTag document totals listed in Table 1, we included documents that were tagged with malignancy mentions that were both strict syntactic parents and biological children of the phrase used. For example, we included articles identified by MTag with the phrase "small-cell lung cancer" within the total for the phrase "lung cancer".

Comparison of these totals between MTag articles and PubMed keyword searching revealed that MTag provided high recall for most malignancies. Interestingly, there are three malignancy mention instances ("carcinoma", "sarcoma", "melanoma") that have more MTag-identified articles than for PubMed keyword searches. This suggests that a more formalized normalization of MTag-derived mentions might assist both with efficiency and recall if employed in concert with the manual annotation procedure currently employed by MEDLINE. Furthermore, MTag's document recall compared quite favorably to exact string matching. Only two of the 25 malignancy mentions yielded less than 60% as many articles via MTag than via PubMed exact string matching ("bone neoplasms" and "lung cancer"). In these two cases, the concept-mapping PubMed search identifies the articles with a broader range beyond the search terms. For example, a PubMed search for the term "lung cancer" identifies articles describing "lung neoplasms", while for "bone neoplams", articles focusing on related concepts such as "osteoma" and "sphenoid meningioma" are identified by PubMed. Generally, MTag recall would be expected to improve further after a subsequent normalization process that maps equivalent phrases to a standard referent.

To assess document-level precision, we randomly selected 100 abstracts identified by MTag each for the malignancies "breast cancer" and "adenocarcinoma". Manual evaluation of these abstracts showed that all of the articles were directly describing the respective malignancies. Finally, we evaluated both the 250 most frequently mentioned malignancies as well as a random set of 250 extracted malignancy mentions from the all-MEDLINE-extracted set. For the frequently occurring mentions, 72.06% were considered to be true malignancies; this set corresponds to 0.043% of all malignancy mentions. For the random set, 78.93% were true malignancies. This suggests that such extracted mention sets might serve as a first-pass exhaustive lexicon of malignancy mentions. Comparison of the entire set of unique mentions with the NCI neoplasm list showed that 1,902 of the 5,555 NCI terms (34.2%) were represented in the extracted literature.

### Availability and Requirements

**Figure 1:**
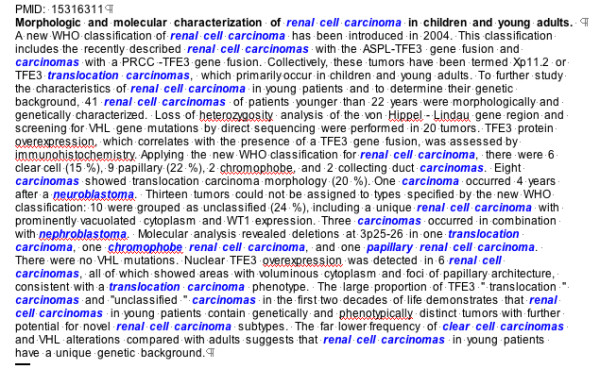
Example of the HTML output of MTag for an annotated abstract [31]. Malignancy type mentions identified by MTag are shown in bold, italicized, and blue text.

MTag is platform independent, written in java, and requires java 1.4.2 or higher to run. The software isavailable without restrictions under the GNU General Public License at . MTag has been engineered to directly accept files downloaded from PubMed and formatted in MEDLINE format as input. MTag provides output options of text or HTML file versions of the extractor results. The text file repeats the input file with recognized malignancy mentions appended at the end of the file. The HTML file provides markup of the original abstract with color-highlighted malignancy mentions, as shown in Figure 1.

## Discussion

We have adapted an entity extraction approach that has been shown to be successful for recognition of molecular biological entities and have shown that it also performs with high accuracy for disease labels. It is evident that an F-measure of 0.83 is not sufficient as a stand-alone approach for curation tasks, such as the *de novo *population of databases. However, such an approach provides highly enriched material for manual curators to utilize further. As was determined by our comparisons with lexical string matching and PubMed-based approaches, our extraction method demonstrated substantial improvement and efficiency over commonly employed methods for document retrieval. Furthermore, MTag appeared to be accurately predicting malignancy mentions by learning and exploiting syntactic patterns encountered in the training corpus.

Analysis of mis-annotations would likely suggest additional features and/or heuristics that could boost performance considerably. For example, anatomical and histological descriptions were frequent among MTag false positive mentions. Incorporation of lexicons for these entity types as negative features within the MTag model would likely increase precision. Our training set also does not include a substantial number of documents that do not contain mentions of malignancy; recent unpublished work from our group suggests that inclusion of such documents significantly impacts extractor performance in a positive manner.

Unlike the first iteration of our CRF model [14], the MTag application required only modest computational effort (several weeks vs. several months) of retraining and customization time (see Methods). To our surprise, the addition of biological features, including an extensive lexicon for malignancy mentions, provided very little boost to the recall rate. This provides evidence that our general CRF model is flexible, broadly applicable, and if these results hold true for additional entity types, might lessen the need for creating highly specified extractors. In addition, the need for extensive domain-specific lexicons, which do not readily exist for many disease attributes, might be obviated. If so, one approach to comprehensive text mining of biomedical literature might be to employ a series of modular extractors, each of which is quickly generated and then trained for a particular entity or relation class. Conversely, it is important to note that the entity class of malignancy possesses a relatively discrete conceptualization relative to certain other phenotypic and disease concepts. Further adaptation of our extractor model for more variably described entity types, such as morphological and developmental descriptions of neoplasms, is underway. However, the finding that biological feature addition provided minimal gain in accuracy suggests that further improvements may be more difficult to obtain than by merely identifying and adding additional domain-specific features. Significantly, challenges in rapid generation of annotations for extractor training, as well as procedures for efficient and accurate entity normalization, still remain.

When combined with expert evaluation of output, extractors can assist with vocabulary building for targeted entity classes. To demonstrate feasibility, we extracted mentions of malignancy for all pre-2006 MEDLINE abstracts. Our results indicate that MTag can generate such a vocabulary readily and with moderate computational resources and expertise. With manual intervention, this list could be linked to the underlying literature records and also integrated with other ontological and database resources, such as the Gene Ontology, UMLS, caBIG, or tumor-specific databases [23-25]. Since normalization of disease-descriptive term lists requires considerable specialized expertise, the role of an extractor in this setting more appropriately serves as an information harvester. However, this role is important, as such supervised lists are often not readily available, due in part to the variability in which phenotypic and disease descriptions can be described, and in part to the lack of nomenclature standards in many cases.

Finally, to our knowledge, MTag is one of the first directed efforts to automatically extract entity mentions in a disease-oriented domain with high accuracy. Therefore, applications such as MTag could contribute to the extraction and integration of unstructured, medically-oriented information, such as physician notes and physician-dictated letters to patients and practitioners. Future work will include determining how well similar extractors perform for identifying mentions of malignant attributes with greater (e.g. tumor histology) and lesser (e.g. tumor clinical stage) semantic and syntactic heterogeneity.

## Conclusion

MTag can automatically identify and extract mentions of malignancy with high accuracy from biomedical text. Generation of MTag required only moderate computational expertise, development time, and domain knowledge. MTag substantially outperformed information retrieval methods using specialized lexicons. MTag also demonstrated the ability to assist with the generation of a literature-based vocabulary for all neoplasm mentions, which is of benefit for data integration procedures requiring normalization of malignancy mentions. Parallel iteration of the core algorithm used for MTag could provide a means for more systematic annotation of unstructured text, involving the identification of many entity types; and application to phenotypic and medical classes of information.

## Methods

### Task definition

Our task was to develop an automated method that would accurately identify and extract strings of text corresponding to a clinician's or researcher's reference to cancer (malignancy). Our definition of the extent of the label "malignancy" was generally the full noun phrase encompassing a mention of a cancer subtype, such that "neuroblastoma", "localized neuroblastoma", and "primary extracranial neuroblastoma" were considered to be distinct mentions of malignancy. Directly adjacent prepositional phrases, such as "cancer <of the lung>", were not allowed, as these constructions often denoted ambiguity as to exact type. Within these confines, the task included identification of all variable descriptions of particular malignancies, such as the forms "squamous cell carcinoma" (histological observation) or "lung cancer" (anatomical location), both of which are underspecified forms of "lung squamous cell carcinoma". Our formal definition of the semantic type "malignancy" can be found at the Penn BioIE website [26].

### Corpora

In order to train and test the extractor with both depth and breadth of entity mention, we combined two corpora for testing. The first corpus concentrated upon a specific malignancy (neuroblastoma) and consisted of 1,000 randomly selected abstracts identified by querying PubMed with the query terms "neuroblastoma" and "gene". The second corpus consisted of 600 abstracts previously selected as likely containing gene mutation instances for genes commonly mutated in a wide variety of malignancies. These sets were combined to create a single corpus of 1,442 abstracts, after eliminating 158 abstracts that appeared to be non-topical, had no abstract body, or were not written in English. This set was manually annotated for tokenization, part-of-speech assignments, and malignancy named entity recognition, the latter in strict adherence to our pre-established entity class definition [27,28]. Sequential dual pass annotations were performed on all documents by experienced annotators with biomedical knowledge, and discrepancies were resolved through forum discussions. A total of 7,303 malignancy mentions were identified in the document set. These annotations are available in corpus release v0.9 from our BioIE website [29].

### Algorithm

Based on the manually annotated data, an automatic malignancy mention extractor (MTag) was developed using the probability model Conditional Random Fields (CRFs) [20]. We have previously demonstrated that this model yields state-of-the-art accuracy for recognition of molecular named entity classes [5,14]. CRFs model the conditional probability of a tag sequence given an observation sequence. We denote that ***O ***is an observation sequence, or a sequence of tokens in the text, and ***t ***is a corresponding tag sequence in which each tag labels the corresponding token with either *Malignancy *(meaning that the token is part of a malignancy mention) or *Other*. CRFs are log-linear models based on a set of feature functions, *f*_*i*_(*t*_*j*_, *t*_*j*-1_, ***O***), which map predicates on observation/tag-transition pairs to binary values. As shown in the formula below, the function value is 1.0 when the tag sequence is *Malignancy*; otherwise (o.w.) it is 0. A particular advantage of this model is that it allows the effects of many potentially informative features to be simultaneously weighed. Consider, for example, the following feature:

fi(tj,tj−1,O)={1.0tj=Malignancy Type,tj−1=Malignancy TypeOj=cancer,Oj−1=lung0O.W.
 MathType@MTEF@5@5@+=feaafiart1ev1aaatCvAUfKttLearuWrP9MDH5MBPbIqV92AaeXatLxBI9gBaebbnrfifHhDYfgasaacH8akY=wiFfYdH8Gipec8Eeeu0xXdbba9frFj0=OqFfea0dXdd9vqai=hGuQ8kuc9pgc9s8qqaq=dirpe0xb9q8qiLsFr0=vr0=vr0dc8meaabaqaciaacaGaaeqabaqabeGadaaakeaacqWGMbGzdaWgaaWcbaGaemyAaKgabeaakiabcIcaOiabdsha0naaBaaaleaacqWGQbGAaeqaaOGaeiilaWIaemiDaq3aaSbaaSqaaiabdQgaQjabgkHiTiabigdaXaqabaGccqGGSaalcqWGpbWtcqGGPaqkcqGH9aqpdaGabeqaauaabeqaciaaaeaacqaIXaqmcqGGUaGlcqaIWaamaeaafaqabeGabaaabaGaemiDaq3aaSbaaSqaaiabdQgaQbqabaGccqGH9aqpcqqGnbqtcqqGHbqycqqGSbaBcqqGPbqAcqqGNbWzcqqGUbGBcqqGHbqycqqGUbGBcqqGJbWycqqG5bqEcqqGGaaicqqGubavcqqG5bqEcqqGWbaCcqqGLbqzcqGGSaalcqWG0baDdaWgaaWcbaGaemOAaOMaeyOeI0IaeGymaedabeaakiabg2da9iabb2eanjabbggaHjabbYgaSjabbMgaPjabbEgaNjabb6gaUjabbggaHjabb6gaUjabbogaJjabbMha5jabbccaGiabbsfaujabbMha5jabbchaWjabbwgaLbqaaiabd+eapnaaBaaaleaacqWGQbGAaeqaaOGaeyypa0Jaee4yamMaeeyyaeMaeeOBa4Maee4yamMaeeyzauMaeeOCaiNaeiilaWIaem4ta80aaSbaaSqaaiabdQgaQjabgkHiTiabigdaXaqabaGccqGH9aqpcqqGSbaBcqqG1bqDcqqGUbGBcqqGNbWzaaaabaGaeGimaadabaGaem4ta8KaeiOla4Iaem4vaCLaeiOla4caaaGaay5Eaaaaaa@8FA2@

This feature represents the probability of whether the token "cancer" is tagged with label *Malignancy *given the presence of "lung" as the previous token. Features such as this would likely receive a high weight, as they represent informative associations between observation predicates and their corresponding labels.

Our CRF algorithm considers many textual features when it makes decisions on classifying whether a word comprises all or part of a malignancy mention. Word-based features included whether a word has been identified as being a malignancy mention by manual annotation of text used as training material. The frequency of each string of 2, 3, or 4 adjacent characters (character n-grams) within each word of the training text was calculated, and the differential frequency of each n-gram within words manually tagged as being malignancy mentions, relative to the overall frequency of these strings in the overall text, was considered as a series of features. Orthographic features included the usage and distribution of punctuation, alternative spellings, and case usage. Domain-specific features comprised a lexicon of 5,555 malignancies and a regular expression for tokens containing the suffix -oma. In total, MTag incorporated 80,294 unique features. All observation predicates, either with or without the biological predicates, were then applied over all labels, applying a token window of (-1, 1) to create the final set of features. The MALLET toolkit [30] was used as the implementation of CRFs to build our model.

### Evaluation

The evaluation set of 432 abstracts comprised 2,031 sentences containing mentions of malignancy and 3,752 sentences without mentions, as determined by manual assessment of entity content. The predicted malignancy mention was considered correctly identified if, and only if, the predicted and manually labeled tags were exactly the same in content and both boundary determinations. The performance of MTag was calculated according to the following metrics: Precision (number of entities predicted correctly divided by the total number of entities predicted), Recall (number of entities predicted correctly divided by the total number of entities identified manually), and F-measure [(2*Precision*Recall)/(Precision+Recall)].

## List of Abbreviations Used

CRF, conditional random field

## Authors' contributions

YJ implemented the algorithm to develop MTag and drafted the manuscript. RM developed the core algorithm and assisted in the implementation. KL developed the software interface. MM supervised the manual annotation for extractor training and testing. SC assisted with the tagging of MEDLINE and analysis of the results. ML oversaw the linguistic aspects of the project. FP developed the theoretical underpinnings of the algorithm and oversaw the computational aspects of the project. RW participated in algorithm design and the manual annotation procedure. PW oversaw the biological aspects of the project, provided overall direction, and finalized the manuscript. All authors read and approved the final manuscript.
